# “They don’t know what it’s really like:” qualitative insights into inpatient cardiac nurses’ perceived workload

**DOI:** 10.1186/s12912-025-03723-4

**Published:** 2025-08-18

**Authors:** Ellen Benjamin, Sarah Romain, Cidalia Vital, Connie Blake, Cynthia Peterson, Joohyun Chung

**Affiliations:** 1 Donna M. and Robert J. Manning College of Nursing & Health Sciences, University of Massachusetts Boston, 100 Morrisey Blvd, Boston, MA 02125 USA; 2 Nursing Research and Holistic Nursing, Baystate Medical Center, 759 Chestnut Street, Springfield, MA 01199 USA; 3 Tan Chingfen Graduate School of Nursing, University of Massachusetts Medical School, 55 Lake Avenue North, Worcester, MA 01655 USA; 4 Elaine Marieb College of Nursing, University of Massachusetts Amherst, 651 N Pleasant St, Amherst, MA 01003 USA; 5 The University of Vermont Medical Center, 111 Colchester Ave, Burlington, VT 05401 USA

**Keywords:** Workload, Nursing, Inpatient, Nursing work, Qualitative research, Personnel staffing and scheduling

## Abstract

**Background:**

Measurements of nursing workload often fail to reflect the complexity of nursing work. Nurses’ perceived workload is shaped by many factors, including patient characteristics, personal, social, organizational, and environmental factors. There is a demonstrated interest in developing more comprehensive nurse workload measurement strategies, but little research has employed qualitative methods to investigate the beliefs and experiences of frontline staff. The purpose of this study was to explore inpatient nurses’ perceptions of their workload and the factors that impact their percieved workload levels.

**Methods:**

This was qualitative study using focus groups. Participants were recruited from the cardiac floors of an urban, academic medical center. A total of 17 nurses participated, including nurses from bedside, charge, educator, and nurse manager roles. Focus group transcripts were analyzed by a team of qualitative investigators using conventional content analysis.

**Results:**

Inpatient nurses’ perceived workload is shaped by their work volume, work attributes, and their ability to complete required tasks while providing meaningful, impactful care. The volume of nursing work is comprised of patient-focused, unit-focused, and institutional-focused tasks. Important work attributes include its perceived urgency, difficulty, alignment to the nurse and unit, interference, unpredictability, and individual nursing burden. Overall, participants expressed deep concern over high workloads that compromise holistic nursing care.

**Conclusion:**

Strategies to more comprehensively measure nurses’ perceived workload should account for the breadth and complexity of nursing work. Nurses should advocate for workload measurement systems that more closely reflect their subjective work experiences.

**Clinical trial registration number:**

Not applicable.

**Supplementary Information:**

The online version contains supplementary material available at 10.1186/s12912-025-03723-4.

## Introduction

Nursing workload has been a prominent focus of healthcare services research due to its impact on patient safety, care quality, healthcare costs, and staff well-being [1–3]. Lower staffing levels are associated with longer lengths of stay, more adverse events, higher risks of hospital mortality, and more frequent instances of missed care [1–4]. Increased nursing workload also corresponds to greater nursing stress, job dissatisfaction, emotional exhaustion, and burnout [5]. Despite extensive research, there is a lack of consensus on the conceptualization and measurement of nursing workload [1, 4, 6, 7].

Historically, nursing workload research has been deeply intertwined with strategies to determine workforce planning and staffing levels [1, 8]. Perhaps due to their staffing focus, existing workload measurement tools offer only limited insight into the complexity of nursing workload. There is now growing interest in developing more comprehensive, holistic workload measures [9]. This process has been hindered by an insufficient understanding of the various factors that shape nurses’ perceived workload [10, 11]. Qualitative research methods are particularly well-suited to address this gap as they can provide deeper insights into the nuanced experiences of nurses. This study explored the following research question: How can inpatient nurses’ perceptions of workload inform more comprehensive workload measures?

## Background

There is an enormous wealth of research investigating nursing workload [1]. The desire to understand and measure nursing work is evident in research dating back to the 1910–1920 s [1, 8]. Studies by Aiken et al., Needleman et al., and Duffield et al. contributed to international awareness of the impact of nursing workload and staffing levels on patient care outcomes [12–14]. The significance of nursing workload has been further underscored by the recent COVID-19 pandemic and ongoing concerns for a severe nursing shortage [15].

Despite its significance, nursing workload has lacked a clear conceptual definition [4, 6, 7]. For decades, the measurement of nursing workload has focused on calculating the total time or number of staff needed to care for patients [6]. These “patient-focused” measures offer a narrow conceptualization of nursing workload [1, 4]. Nursing workload has been equated to measures of nursing intensity, patient acuity or severity, patient dependency, nurse-patient ratios, and calculations of nursing hours per patient day [1, 6]. Prominent examples of patient-focused workload measures include the Therapeutic Intervention Scoring System (TISS), the Nursing Activities Score (NAS), and the Nine Equivalents of Nursing Manpower Use Score (NEMS) [4, 16, 17].

The TISS, introduced in 1974 by Cullen et al., identified 57 therapeutic interventions performed in the critical care setting. Such interventions ranged from the need for ECG monitoring, IV antibiotics, or a urinary catheter to cardiac arrest, balloon tampinade, and controlled ventilation [18]. Although the TISS was originally intented to quantitatively differentiate patients’ severity levels, it was quickly applied to evaluate staffing and resource allocation [18]. The tool was subsequently was revised to 76 and, ultimately, 28 items [19, 20]. In 1997, Miranda et al. used the TISS to derive a new, simplified intervention scoring system called the NEMS that contained only 9 items [21]. Later, in acknowledgement that the TISS and NEMS did not accurately account for nurses’ time spent caring for individual patients, Miranda et al. proposed the NAS [22]. The NAS encompasses nursing care activities that demand nursing care time irrespective of patients’ severity levels, such as hygiene, mobility, and rehabilitation. Despite these advancements, studies show that these instruments capture only about 50–80% of the total nursing care time [22].

In contrast to patient-focused workload measures, perceived workload measures aim to describe the subjective experiences of nurses. This more expansive conceptualization of workload reflects the physical, emotional, temporal, and cognitive burdens of work and is commonly measured using the NASA-TLX [4]. Notably, the TISS, NAS, and NEMS are poorly or only moderately correlated with measures of perceived workload [3, 4, 16, 23]. The misalignment between patient-focused and perceived workload measures suggests that existing staffing methodologies fail to adequately reflect nurses’ subjective experience of their work demands.

Perceived nursing workload is shaped by a broad range of personal, patient, social, organizational, and environmental factors [2, 4]. Such factors may include patient turnover, patient and family communication, teamwork, interruptions, patient isolation status, shift timing, documentation burdens, unscheduled activities, work variability, and nurse experience levels [2, 3, 9, 11, 24, 25]. Perceived workload measures typically require working nurses to complete subjective assessments during or immediately after their shift. Identifying specific, measurable workload indicators within electronic health record or administrative data could reduce this burden on working staff [9–11, 24, 25], but empirical explorations of workload indicators remain limited and scholars have argued this research is still in its infancy [10, 11].

A qualitative study can address this knowledge gap by providing rich, in-depth insight into nurses’ perceptions of their workload. By providing context to existing data, qualitative research may also enhance staffing methodologies and help them better reflect the real-world experiences of nurses. Ultimately, this is crucial for developing more comprehensive, effective staffing and nurse-patient assignment strategies that improve nurse well-being, reduce burnout, and enhance patient care outcomes. The aim of this study was to explore inpatient nurses’ perceptions of their workload and the factors that impact their perceived workload levels.

## Methods

### Design and theoretical framework

This study was a qualitative focus group study of inpatient cardiac nurses. The study was guided by Jackson et al.’s integrated framework of nursing work [26]. Jackson et al. proposed that nursing work is complex, multifaceted, and is often hidden from view. Their framework categorizes nursing work into four distinct types of labor: physical, emotional, cognitive, and organizational [26]. The study purpose, interview guide, and data analysis were all informed by this broad conceptualization of nursing work. This manuscript was guided by the COnsolidated criteria for REporting Qualitative research (COREQ) reporting checklist.

### Study setting and recruitment

Inpatient registered nurses were purposefully recruited from three cardiac inpatient units at a single hospital located in New England. The hospital was an urban, academic medical center with more than 700 inpatient beds. The inpatient units included both acute and intermediate level care beds that serve patients with chronic and acute cardiac conditions, including congestive heart failure, myocardial infarction, and post-cardiac surgical and interventional procedures. These floors also routinely care for overflow from other inpatient medical-surgical and intermediate units.

Recruitment was conducted through word of mouth and email outreach. Eligible participants included registered nurses in staff, charge, educator, and nurse manager roles. The inclusion criteria were as follows: nurses who had been employed in the units for a minimum of six months, were proficient in English, and expressed willingness to participate. No relationships were established with study partcipants prior to the study commencement. Participants were made aware of the purpose of the study and the investigators’ interest in better understanding inpatient nusing workload.

### Data collection

Five focus groups were conducted remotely over Zoom between June and November of 2024. A total of 17 participants took part, with each group comprising 3 to 4 participants. The decision to use smaller focus groups was intentional, as smaller groups are well-suited for exploring emotionally charged or sensitive topics [27]. In this study, participants openly shared their experiences related to workload, and the smaller group format supported more personal, in-depth discussions. A semi-structured interview guide was used to direct questioning and included probes to elucidate greater depth of response. See sample in Supplementary Materials.

All study authors participated in conducting and observing focus groups. Authors include four PhD prepared nurse scientists employed in academic and/or settings (EB, CV, CP, JC), one nurse researcher with an EdD degree (CB), and one doctoral student also employed as a clinical research nurse (SR). All are female registered nurses. Meetings lasted approximately 60 min and were audio and video-recorded and transcribed. Authors also took field notes to record observations about participants’ reactions, nonverbal behavior, tone of voice, and personal thoughts.

Data collection continued until data saturation was reached, defined as the point at which new data was largely redundant with previously collected data [28]. Data saturation was assessed by three investigators (EB, SR, CV) after the analysis of each focus group, beginning after the second focus group. Saturation was determined to be reached after the fifth focus group, when little novel data were collected and researchers agreed that interviews were robust.

### Data analysis

Data analysis was conducted concurrently with data collection. Data were analyzed using conventional qualitative content analysis, as described by Hseih & Shannon [29]. Qualitative content analysis is a form of data analysis used to classify large volumes of textual data, such as focus group transcript data, into discrete categories of similar meanings with the purpose of describing a phenomenon. In conventional content analysis, coding and category development are performed inductively, such that categories emerge from the data [29].

To familiarize themselves with the data, four research team members independently listened to focus group recordings, read observational notes recorded during focus groups, and immersed themselves in focus group transcripts. Coding and preliminary category development were conducted independently by four team members (EB, SR, CV, CP) in Microsoft Word and NVivo12 Software. Coding began with initial, line-by-line descriptive and in vivo coding and progressed to additional rounds of coding larger segments of data. Codes were compared and sorted according to their similarities, patterns, and relationships to cluster codes into meaningful emergent categories. Memo-writing was used to deepen data analysis, organize emergent categories and sub-categories, and prompt reflexivity.

Three research team members (EB, SR, CV) met collectively over several days to discuss initial coding schemes and emergent categories. Identification of final categories and sub-categories were based on discussion and consensus among these three analysts. Definitions for each sub-category and category were clarified, and supporting evidence was organized into an audit trail.

### Ethical considerations

This study was approved by the University of Massachusetts Amherst Institutional Review Board and the IRB of the participating health system. Participants were provided with an electronic informed consent document and indicated their consent by checking a checkbox and typing their name.

### Trustworthiness

Study trustworthiness was supported through the use of multiple data analysts and a consensus approach to final category development. Nurses in diverse roles were purposefully recruited to capture a greater breadth of nursing experience and negative case analysis was intentionally pursued. Investigators closely adhered to an articulated analysis methodology and engaged in memo-writing to promote reflexivity. Analysis transparency and auditability were promoted through the use of qualitative analysis software. Study findings are supported by an audit trail and rich data evidence.

## Findings

### Characteristics of participants

A total of 17 nurses participated in 5 focus groups. No participants dropped out. Nurses varied by years of experience and educational level. Participant demographics are presented in Table 1.

**Table 1:** Demographic characteristics

		*n*	%	
Unit	Unit A	2	11.8%	
	Unit B	4	23.5%	
	Unit C	10	58.8%	
	Preferred not to answer or missing	1	6.9%	
Role	Staff nurse	6	23.5%	
	Charge nurse	6	23.5%	
	Nurse manager or educator	4	23.5%	
	Other	1	6.9%	
Age	20–29	2	11.8%	
	30–39	9	52.9%	
	40–49	1	6.9%	
	50–59	3	17.7%	
	60+	1	6.9%	
	Preferred not to answer or missing	1	6.9%	
Gender	Female	13	76.5%	
	Male	3	17.7%	
	Preferred not to answer or missing	1	6.9%	
Level of education	Associate degree	2	11.8%	
	Bachelor’s degree	12	70.6%	
	Master’s degree	3	17.7%	

### Categories

**Fig. 1:**
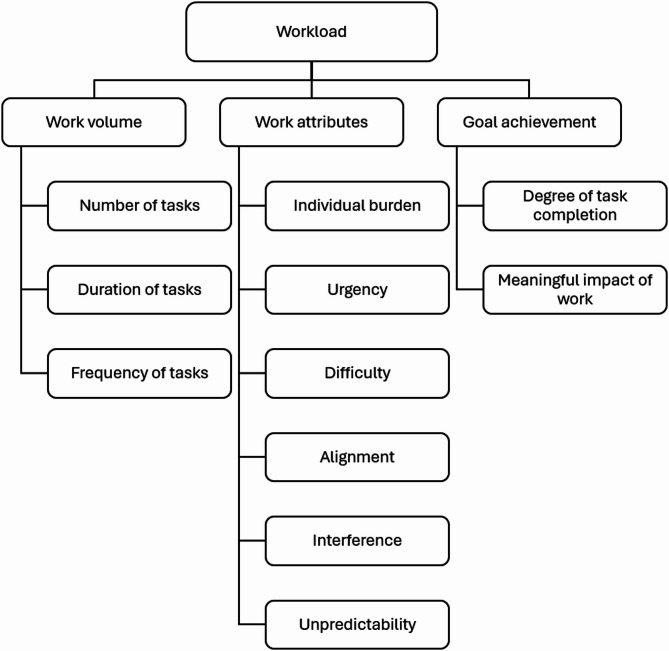
Inpatient nurses’ perceptions of workload

Three overarching categories emerged to describe inpatient nurses’ perceptions of workload: work volume, work attributes, and goal achievement. Major categories and subcategories are discussed below and summarized in Fig. 1. More detailed categories, subcategories, codes, and supporting data are presented in Table 2.

### Work volume

Nurses’ perceived workload depends on the total volume of work required each shift. This work volume arises from the number, duration, and frequency of required tasks. Here, the word “task” is used to reflect the broad nature of nursing work, encompassing duties beyond what is traditionally conceptualized as nursing interventions or care activities.

#### Number of tasks

First, work volume is shaped by the number of tasks that nurses perform. Participants described a great breadth of tasks that all contribute to their overall workload, including tasks that are patient-focused, unit-focused, or institution-focused.

Patient-focused tasks encompass a wide range of nursing activities that directly serve patient care needs. Ordered care tasks that appear within the electronic health record are just one component of this patient-focused work. Nurses are also responsible for providing patient hygiene and comfort; developing knowledge of their patient through assessments, reports, and chart review; communicating with patients and visitors; planning and advancing patients’ care trajectories; advocating and managing escalations in care; and maintaining patient safety.

*You can look at the patient load and look at what the orders are. And that’s just*,* at a minimum*,* what is required. (Participant A1)*

Unit-focused tasks describe the work that nurses perform to support the staff and functioning of their unit. Such work includes staffing and scheduling, helping peers, mentoring, and providing training or orientation for new staff. Of these, participants emphasized the demands of training as especially burdensome.

*With precepting that’s exhausting…. And they’re our responsibility on top of our six or seven patients*,* and it’s just exhausting. (Participant E2)*

Institution-focused tasks are required by the hospital but do not directly serve individual patients or units. Institutional tasks include reporting, data gathering, attending staff meetings, and performing administrative work, such as conducting chart reviews, auditing, and sending emails. Institutional work also encompasses physical logistics and resource management work, such as managing patient bed assignments, cleaning rooms, changing bed linens, gathering supplies, and managing equipment.

*Any time there’s any kind of a new process*,* or a new audit*,* or… we have to start evaluating certain things*,* and collect data…. we try to encourage greater involvement from the staff to try and assist with some of those things. (Participant A3)*

#### Duration of tasks

Work volume also depends on the duration of nursing tasks. Some tasks can be completed very quickly while others consume large proportions of a nurse’s shift. Examples of time-consuming tasks include documenting, managing patient deteriorations, and completing medication passes at the beginning of each shift. Task duration also varies according to the complexity and personality of each patient.

*When you have patients who on paper…look fine [but] they have 35 pills*,* and they have to take them all one at a time. (Participant B3)*

#### Frequency of tasks

Work volume is also impacted by task frequency. Many nursing tasks must be performed repeatedly over the course of a single shift. Examples of frequently repeated tasks include blood sugar assessments, surgical site checks, pain medication administrations, and vital sign assessments. Nurses who experience high patient turnover within their assignment must also repeat tasks for newly arrived patients.

*A lot of it depends on the frequency of their interventions. (Participant A3)*

### Work attributes

In contrast to work volume, work attributes describe the quality and nature of nursing work. Nurses’ perceptions of their workload vary according to the pace, rhythm, and complexity of their work. Six unique sub-categories emerged: urgency, individual burden, difficulty, alignment, interference, and unpredictability.

#### Urgency

Facing high volumes of required tasks, nurses must prioritize their time and effort. Nursing workload is impacted by the perception of how urgently each task must be performed. A sense of urgency is often felt when patients are unstable or very ill. Nurses may also experience urgency from the desire to adhere to their patient’s schedule, such as administering scheduled medications or completing diagnostic tests on time.

*You try to stay on track as much as possible*,* because certain things have to be done*,* and they have due dates*,* and they must be done*,* no matter what. (Participant A1)*

#### Individual burden

Individual work burden describes the degree to which required work falls on any individual nurse. Units with staff in supportive roles, such as charge nurses, float nurses, or nursing assistants, are able to distribute bedside nursing work across a wider staffing team. Nurses in units with poor staffing experience a higher individual burden of work.

*Staffing is a huge…I do notice once we tip the scale with that extra patient*,* people really start to get on edge*,* ‘cause it’s just not safe. (Participant C2)*

A sense of individual burden is also shaped by nurses’ ability to take breaks. Nurses unable to take breaks may perceive their work as more continuous, unrelenting, and unreasonable.

*You’re not allowed time to*,* kind of like*,* to process anything that just happened. You’re*,* kind of*,* you just gotta jump back in and pick up where you left off. (Participant E2)*

#### Difficulty

Work difficulty describes how physically, mentally, and emotionally challenging nurses find their work. For inpatient cardiac nurses, work becomes more difficult when they are asked to float outside of their traditional unit, are unfamiliar with the patient population, face highly complex or unfamiliar procedures, or must deal with changing equipment or processes. Work is also made more difficult by the constant need to multi-task. Nurses described this idea using words such as “*juggling*,” managing “a *whole lot of moving parts*,” “*scrambling*,” and being “*spread thin*.”

Participants were also candid about the emotional difficulty of their work. Nurses recounted feelings of exhaustion, failure, inadequacy, powerlessness, burnout, resignation, and being overwhelmed. Many aspects of nursing work can be emotionally challenging, but participants tended to emphasize the significance of negative interactions with visitors and their own colleagues.

*It’s a terrible*,* helpless*,* horrible feeling to be berated by providers and other people. (Participant E1)*

Although participants did not highlight the physical difficulty of their work, they did note that nursing work can be exhausting due to the demands of lifting, turning, and walking.

*On an inpatient busy telemetry floor*,* like it’s tiring*,* your feet hurt….it’s physically exhausting in the way that you’re truly not sitting down. The only time you sit down is to chart. (Participant E1)*

#### Alignment

Work alignment describes the degree to which a nurse’s work is appropriate for their nursing unit. Hospital units are staffed according to patients’ acuity and care needs, and patients who are incorrectly assigned to lower acuity floors demand disproportionately high amounts of nursing time. Participants reported frequent occurrences of mismatches between patients’ clinical acuity and their assigned level of care.

*You have five regular tele patients and two intercare patients. The assignment isn’t adjusted for that. (Participant E1)*

Alignment also reflects whether patients are well-matched to their nurse’s personality and experience level. Nurses vary in their strengths and weaknesses, including their level of clinical expertise, ability to manage emergencies, organizational knowledge, and interpersonal skills.

*If you go in there and you rush*,* somebody might have anxiety. Then you’re just gonna really break the relationship right from the beginning. Whereas if you needed to spend that*,* you know*,* extra TLC and explain to them*,* and make them feel like they can trust you*,* and all that stuff. (Participant C2)*

#### Interference

Task interference describes the extent to which a nurse’s ability to complete their work feels impeded. Interruptions and multiple competing demands can distract nurses from being able to carry out their intended tasks.

*Being a floor nurse- because you’re pulled in a million different directions at any given time during a shift. (Participant D2).*

Nurses may also feel a sense of interference from patient preferences or institutional priorities. For example, nurses may have to alter their medication passes to accommodate patients who want their medications delivered at a different time. Also, institutional pressure to expedite patient turnover often feels at odds with nurses’ own goals and workflows.

*You know you’ve got management saying*,* ‘Hey*,* by the way*,* get this patient out*,* go give report on that.’ Well*,* so do you want me to give report*,* or do you want me to give medication? (Participant B4)*

#### Unpredictability

Nurses’ perceived workload is influenced by feelings of unpredictability. Participants described their work as “*chaotic*” and “*hard to calibrate.”* For inpatient cardiac nurses, every shift is different and assignments turn over frequently. Patient needs can also suddenly change, such as when patients acutely deteriorate, fall, or experience a rapid change in behavior. This unpredictability means that even simple care tasks may be unexpectedly time-consuming and nurses have limited ability to anticipate their work demands.

*Our patients are pretty critical… that can change in a blink of an eye. So*,* what you think might be the most stable patient- for instance*,* we had a patient that was independent*,* right? We call them walkie talkies. Guess who fell? (Participant C2)*

### Goal achievement

Last, nurses’ perceived workload is shaped by their ability to achieve their work goals. Nurses alluded to two distinct goals: the ability to complete assigned tasks and the ability to provide meaningful, impactful care.

#### Degree of completion

Nurses strive to meet an expected standard of care. Nurses expect to complete their ordered care tasks and adequately meet their patients’ hygiene, activity, comfort, socioemotional, and educational needs. Nurses also hope to finish their documentation by the end of their shift.

*I would say*,* the thing that gets omitted is that teaching moment*,* that*,* that sitting down with your patient and actually having a conversation with them. (Participant A2)*

#### Meaningfulness of work

Nurses’ sense of their workload is also impacted by the perceived meaningfulness of their work. In addition to completing required tasks, participants described the desire to spend time with their patients, feel a sense of connection, provide extra comfort, and include more holistic nursing care.

*There are a lot of tasks that might feel more rewarding or fulfilling if I felt like I was able to actually have the time to do them … actually spend time with them and talk to them*,* come and create some form of connection for the day and feel as though I’m really there for them. (Participant E3)*

Notably, nurses report that their ability to provide meaningful work is often in tension with their ability to fully complete their assigned tasks. Participants described struggling to balance these two important priorities throughout their shift.

*Even when I connect*,* it’s stressful knowing that- because you can’t really connect*,* right?…you realize*,* like*,* ‘Shoot*,* I just spent 15 minutes connecting with this patient*,* and now I’m behind on my actual nursing tasks.’ It’s a little sad. It’s sad. (Participant E2)*

**Table 2:** Major and minor categories, codes, and supporting evidence

Major Category	Minor Category	Code	Participant Quote
Work Volume	Number of tasks	Patient-focused tasks	“So it’s all based on your order set of what you need to do for your patient” (P. C2)
		Unit-focused tasks	“As a charge nurse it gets to be even more complicated, because at that hour, you are also getting the staffing, you are scrambling to try to make an assignment.” (P. B3)
		Institution-focused tasks	“In measuring the workload, there’s also a lot of meetings that we do have to attend that kind of pull you away…because you’re sitting in on a meeting and you’re not doing these other tasks that need to be done” (P. D2)
	Duration of tasks	Time-consuming tasks	“I start with my morning medications that usually takes me from the start of the shift, until sometimes I’m running my morning med pass until 11 o’clock.” (P. B4)
		Time-consuming patients	“I spent, you know, at least two, two and a half hours on that RRT in total for the stroke.” (P. E2)
	Frequency of tasks	Frequency of orders/ procedures	“Your other two can be vascular surgery, which are extremely pain med heavy, having frequent such as Q2 hours IV push Dilaudid and Q3 PO. So if you’re doing the math, the amount of time that you have to be in these rooms…is just astronomical” (P. E1)
		Patient turnover	“Yesterday *[nurse]* discharged 16 patients…they got 15 patients back. Most of it happened within 3 hours. You cannot tell me that can be done in a safe manner. If you try to tell me that, I want to see you do it with us.” (P. B3)
Work Attributes	Individual burden	Nurse-patient ratio	“I mean, if you have enough nurses to have a 5-to-1 patient ratio workloads’ better. If you don’t, and 7-to-1, it’s just- that’s the biggest factor.” (P. C1)
		Level of teamwork	“I just think it’s really just based on the relationship you have with your coworkers really.” (P. C1)
		Staff support	“You also need to make sure that all those patients are up in the chair, otherwise it falls on the nurse, even though that’s a PCT task.” (P. E1)
		Ability to take breaks	“I try and stress that with the staff to make sure that they take a break, because they just feel it’s like,’ Oh, it’s just gonna be all piled up when I get back.’” (P. A3)
	Urgency	Patient acuity	“The first thing is acuity, what’s going on with your patients? And what do they need, based on medical needs? (P. A2)
		Schedued tasks	“I think we have to be very time oriented. You know, my meds are due at this time.” (P. C1)
	Difficulty	Cognitive difficulty	“It’s a lot of juggling and trying to multitask, and sometimes it feels like I’m just chasing my tail.” (P. D1)
		Emotional difficultly	“We had another patient… [who was] uncooperative, very demanding…turning staff against each other… There was a lot of stress around that situation, mentally, emotionally, physically. That definitely put a damper on staff morale and was very heavy in the workload, and nobody ever wanted that patient because they were not a nice person.” (P. B4)
		Physical difficulty	“We have a patient on our floor now, that’s 850 pounds; that’s a very large person to have to roll back and forth” (P. C2)
	Alignment	Nursing expertise	“We may have a patient who may have more complicated issues going on, and, you know, a more experienced nurse may be better to assign that patient.” (P. A1)
		Nursing personality	“You cannot assign this patient to me. It is not therapeutic for him. It’s not therapeutic for me…Ultimately we were able to switch it. It went really well, he responded well to the other *[nurse*].” (P. B3)
		Patient appropriateness for unit	“You need to go to a higher level of care, and that’s 1 of 6 that ends up taking up a lot of your time and just feel like you’re behind for the rest of the shift, trying to catch up and make sure the other 5 patients at the care that they need.” (P. C1)
	Interference	Interruptions	“I [try to] accomplish something for all the different tasks I have to do …. which can be quite difficult, because at any time you can be interrupted by a provider, a nurse with a question…” (P. D1)
		Multiple competing tasks	“There’s a whole lot of moving parts to that wheel that we have to sort of account for at all times.” (P. B4)
		Patient preferences	“You’re letting them sleep. They need sleep. I mean, all studies have shown that that’s basic for healing.” (P. B3)
		Organizational priorities	“[*The hospital unit]* needs open beds for multiple reasons. So, you’re kind of like rushing around, ‘Yeah, we can’- You know, ‘We’ll take this out. We can shut this off and we’ll send them up.’” (P. E1).
	Unpredictability	Variability across patients	“It can change minute to minute.” (P. C2)
		Variability across time	“Every shift is kind of different.” (P. A2)
Goal achievement	Degree of task completion	Meeting standard of care	“But then you have other [nights], where they’re sleeping… You can get all your work done by 12 o’clock, 2 o’clock.” (P. B3)
		Missed or omitted care	“You just got to jump back in and… catch up on pain meds for everyone else, and pages that you might have missed about other patients, and it’s just a lot.” (P. E2)
	Meaningfulness of work	Connecting with patients	“Being able to go there and connect with them and let them know that I’m here… has made a big difference.” (P. D1)
		Holistic nursing practice	“I can’t tell you how many times I make sure that I lotion the patient’s legs before bed, because when I see those Lasix legs that are super dried and scaly, I can’t stand it. And they seem to really appreciate it, too, and it helps them relax.” (P. B3)
		Unrewarding work	“We’re expected to properly chart on every patient. And that is tedious and painful in its own way.” (P. E2)

## Discussion

The purpose of this study was to explore how inpatient nurses perceive their workload and the factors that shape their workload levels. This paper adds to ongoing efforts to identify potential nursing workload indicators [9–11, 24, 25] by considering the expertise of frontline nursing staff and nurse leaders. Emerging research is consistent with the understanding that nurses’ perceived workload is shaped by diverse human and system factors [9–11, 24, 25]. In alignment with our findings, studies have demonstrated that nurses’ perceived workload is significantly related to medical urgency, staffing resources, documentation burdens, unpredictable activities, interdisciplinary collaboration, peer support, and patient turnover [10, 11, 24]. Although scholars have begun to identify individual workload indicators, this study is the first, to our knowledge, to offer a comprehensive model of inpatient nurses’ perceived workload that is grounded in rich, empirical qualitative data.

Through five focus groups with 17 nurse participants, three categories emerged to capture nurses’ perceptions of workload: volume of work, attributes of work, and ability to achieve care goals. Much of nursing work focuses on directly addressing patient care needs. This volume of work is determined by the number, duration, and frequency of required patient care tasks. While some patients require a few brief tasks, other patients require many tasks that are repetitive and time-consuming. Prominent workload measurement systems, including the Therapeutic Intervention Scoring System (TISS), Nursing Activities Score (NAS), and Nine Equivalents of Nursing Manpower Use Score (NEMS), are well-suited to objectively quantify such patient-focused tasks [18–22]. However, these instruments do not comprehensively capture the breadth of nurses’ work volume [22], nor fully reflect the complexity, variability, and relational aspects of nursing work.

Nurses’ total work volume not only reflects patient-focused tasks, but also includes unit and institutional responsibilities [6, 7, 30]. There have been important efforts to measure these non-patient aspects of nursing workload, such as the development of the RAFAELA system in Finland [30], yet many workload management systems are poorly equipped to quantify the demands of scheduling and staffing, training, mentorship, administrative work, documentation, auditing, staff meetings, or resource management. Among these, participants reported that documentation and staff training are especially demanding. Recent high rates of staff turnover and a lack of experienced nurses have increased the burdens of training and mentorship on a shrinking pool of qualified preceptors [31]. This changing nursing workforce heightens the need to more fully consider nurses’ unit and institutional responsibilities as important elements of their total work volume.

In addition to their work volume, inpatient nurses’ perceived workload is shaped by the characteristics and nature of that work. Overall, study participants raised concerns over work environments that are challenging, fast-paced, and contain insufficient resources and support. Inpatient nurses struggle to meet many, urgent, competing priorities, including caring for patients that are poorly matched to their unit’s acuity level and nursing experience. These more subjective perceptions of nursing workload are not routinely captured in staffing methodologies, but they are critical to more fully understand, monitor, and compare workload levels in order to improve nurses’ work performance and well-being [4].

The six work attributes described in this paper echo many of the workload dimensions articulated by Carayon and Alvarado in their discussion of critical care nurses [32]. Carayon and Alvarado write that critical care nurses’ workload is comprised of multiple elements, including physical, cognitive, and emotional workloads. Like current study participants, they argue that workload is also determined by the quantity and difficulty of work, work variability, and the burdens of time pressure and temporal constraints [32]. Whereas Carayon and Alvarado delineate the dimensions that comprise nursing workload, the attributes we describe─ individual burden, urgency, difficulty, alignment, interference, and unpredictability─ reflect nurses’ subjective experience of their work. Notable contributions of this study are the importance of work interference and work alignment. Although interruptions and nursing expertise are often recognized for their potential impact on perceived workload [11, 24, 32–34], other findings, such as interference from conflicting patient and organizational priorities, the role of nurses’ personalities, and patient-to-unit misalignments, are poorly understood. Ivziku et al. have demonstrated that inpatient nurses report higher perceived workload when they care for patients with a broader range of medical specialties [11, 24], but more research is needed to investigate the prevalence and impact of misalignments between nursing units and patients’ assigned level of care.

Finally, this study found that nurses’ perceived workload is impacted by their ability to meet their care goals. This finding resembles a dimension of the widely used NASA-Task Load Index, which assesses workers’ satisfaction with their job performance [4], but more clearly articulates the goals and perspectives of inpatient nurses. This category also highlights the inherent tensions and tradeoffs of nursing work. Inpatient nurses desire a sense of connection with their patients that strengthens their nurse-patient relationship and allows them to provide greater comfort, socioemotional support, and holistic care. Unfortunately, when faced with the need to ration care, nurses are often unable to adequately meet the emotional, educational, and comfort needs of their patients [35]. Some scholars have argued that the realities of resource scarcity and efficiency-driven healthcare systems threaten the fundamental values and aims of nursing practice [35]. It is critical that nurses play an active role in defining and determining staffing workloads in a manner that is consistent with our holistic goals of care. Nurses and patients both suffer when extreme workloads threaten the quality and richness of nursing work [35].

### Limitations

This qualitative study was initially planned as the first phase of a larger mixed-methods study aimed at identifying nursing workload indicators. However, the second phase, which involved collecting quantitative workload data, was not completed due to challenges in obtaining the necessary data. As a result, the study’s findings are limited to qualitative insights, and the inability to combine these with quantitative data restricts the ability to triangulate findings.

Additionally, the sample was purposefully recruited from three cardiac units at a single hospital. While effort was taken to include nurses from a variety of floors and roles, including floor nurses, educators, and nurse managers, the findings may not fully represent the broader range of inpatient nurses’ perceptions of workload. Thus, the experiences captured in this study might not reflect the complete spectrum of workload perceptions across the broader inpatient nursing population. Furthermore, self-selection bias may be present, such that nurses willing to participate in this study may have felt more strongly or been more critical of their workload levels than the broader inpatient nursing population.

Saturation was determined based on the redundancy of collected data, a definition that may lead to reaching saturation at a relatively early phase of collection and analysis [28]. It is possible that additional focus groups or larger focus groups with more participants may have yielded more workload categories not described in this manuscript.

### Recommendations for further research

The central contribution of this paper is a model based on qualitative analysis that describes inpatient nurses’ perceptions of their workload. This model provides a framework to better understand, organize, and identify varied workload indicators. Measurement approaches that aim to more comprehensively capture nursing workload should quantify the volume of nursing tasks, the attributes of nursing work, and nurses’ ability to achieve their holistic care goals. To advance this research, specific workload indicators should be defined within each workload domain. New instruments may be needed to measure all aspects of nurses’ perceived workload, such as perceived interference and unpredictability.

### Implications for policy and practice

Focus group participants emphasized the ongoing need for targeted strategies to reduce inpatient nursing workloads. Nursing administration and policy efforts may use this qualitative model to develop a multifaceted workload management approach. Interventions should focus on reducing the volume of patient-related, unit-based, and institutional tasks while also considering the complex social, organizational, and temporal factors involved. Practical approaches may include minimizing unit and institutional responsibilities, alleviating training burdens, diminishing work interruptions, promoting teamwork, increasing staffing levels, reducing unpredictability, and ensuring better alignment between patients, hospital units, and nursing staff. Enhancing technology and optimizing workflows may also significantly lessen administrative burdens. Policy initiatives should advocate for safe and healthy working environments, including appropriate nurse-to-patient assignments, and actively involve nurses in decision-making related to workload management.

## Conclusion

Inpatient nurses report working in challenging environments with high workloads. Nurses’ perceived workload arises from the characteristics and care needs of their patients, alongside their unit and institutional responsibilities. Nurses’ workload perceptions are also shaped by the urgency, perceived difficulty, unpredictability, alignment, interference, and individual burden of their work. Workload levels are perceived as unreasonable when nurses are unable to fully complete their care tasks and provide meaningful, impactful patient care. Researchers interested in more holistically measuring inpatient nursing workload may consider this qualitative model as a basis for identifying perceived workload indicators.

## Supplementary Information

Below is the link to the electronic supplementary material.

Supplementary Material 1

Supplementary Material 2

## Data Availability

The datasets generated and/or analysed during the current study are not publicly available due concern for participant privacy but excerpts are available from the corresponding author on reasonable request.

## References

[1] Griffiths P, Saville C, Ball J, Jones J, Pattison N, Monks T. Nursing workload, nurse staffing methodologies and tools: a systematic scoping review and discussion. Int J Nurs Stud. 2020;103:103487. 31884330 10.1016/j.ijnurstu.2019.103487 PMC7086229

[2] Swiger PA, Vance DE, Patrician PA. Nursing workload in the acute-care setting: A concept analysis of nursing workload. Nurs Outlook. 2016;64(3):244–54. 26944266 10.1016/j.outlook.2016.01.003

[3] Nasirizad Moghadam K, Chehrzad MM, Reza Masouleh S, Maleki M, Mardani A, Atharyan S, et al. Nursing physical workload and mental workload in intensive care units: are they related? Nurs Open. 2021;8(4):1625–33. 33596333 10.1002/nop2.785 PMC8186696

[4] Hoonakker P, Carayon P, Gurses A, Brown R, McGuire K, Khunlertkit A, et al. Measuring workload of ICU nurses with a questionnaire survey: the NASA task load index. IIE Trans Healthc Syst Eng. 2011;1(2):131–43. 22773941 10.1080/19488300.2011.609524 PMC3388621

[5] Maghsoud F, Rezaei M, Asgarian FS, Rassouli M. Workload and quality of nursing care: the mediating role of implicit rationing of nursing care, job satisfaction and emotional exhaustion by using structural equations modeling approach. BMC Nurs. 2022;21(1):273. 36209155 10.1186/s12912-022-01055-1 PMC9548180

[6] Alghamdi MG. Nursing workload: a concept analysis. J Nurs Manag. 2016;24(4):449–57. 26749124 10.1111/jonm.12354

[7] Morris R, MacNeela P, Scott A, Treacy P, Hyde A. Reconsidering the conceptualization of nursing workload: literature review. J Adv Nurs. 2007;57(5):463–71. 17284279 10.1111/j.1365-2648.2006.04134.x

[8] Clopton EL, Hyrkäs EK. Modeling emergency department nursing workload in real time: an exploratory study. Int Emerg Nurs. 2020;48:100793. 31732454 10.1016/j.ienj.2019.100793

[9] Chen Y, Chan CW, Dong J, Jackson EM, Yip NH, Rossetti SC. Beyond order-based nursing workload: A retrospective cohort study in intensive care units. J Nurs Scholarsh. 2024;56(5):687–93. 38736177 10.1111/jnu.12979

[10] Oppel EM, Mohr DC. Multilevel analysis exploring the relative importance of workplace resources in explaining nurses’ workload perceptions: are we setting the right focus? Health Care Manage Rev. 2021;46(2):E8–17. 33630510 10.1097/HMR.0000000000000285

[11] Ivziku D, Ferramosca FMP, Filomeno L, Gualandi R, De Maria M, Tartaglini D. Defining nursing workload predictors: a pilot study. J Nurs Manag. 2022;30(2):473–81. 34825432 10.1111/jonm.13523 PMC9300160

[12] Aiken LH, Clarke SP, Sloane DM, Sochalski J, Silber JH. Hospital nurse staffing and patient mortality, nurse burnout, and job dissatisfaction. JAMA. 2002;288(16):1987–93. 12387650 10.1001/jama.288.16.1987

[13] Needleman J, Buerhaus P, Mattke S, Stewart M, Zelevinsky K. Nurse-staffing levels and the quality of care in hospitals. N Engl J Med. 2002;346(22):1715–22. 12037152 10.1056/NEJMsa012247

[14] Duffield C, Diers D, O’Brien-Pallas L, Aisbett C, Roche M, King M, et al. Nursing staffing, nursing workload, the work environment and patient outcomes. Appl Nurs Res. 2011;24(4):244–55. 20974086 10.1016/j.apnr.2009.12.004

[15] MacPhee M, Havaei F, Bookey-Bassett S, Neumann WP, Qureshi SM, Greig MA, et al. Commentary on the past, present, and future of nursing workload research. Nurs Res Rev. 2024;14:59–67.

[16] Wallet F, Bonnet A, Thiriaud V, Caillet A, Piriou V, Vacheron C-H, et al. Weak correlation between perceived and measured intensive care unit nursing workload: an observational study. J Nurs Care Qual. 2024;39(3):E39–45. 38780353 10.1097/NCQ.0000000000000774

[17] Racy S, Davidson PM, Peeler A, Hager DN, Street L, Koirala B. A review of inpatient nursing workload measures. J Clin Nurs. 2021;30(13–14):1799–809. 33503306 10.1111/jocn.15676

[18] Cullen DJ, Civetta JM, Briggs BA, Ferrara LC. Therapeutic intervention scoring system: a method for quantitative comparison of patient care. Crit Care Med. 1974;2(2):57–60. 4832281

[19] Reis Miranda D, de Rijk A, Schaufeli W. Simplified therapeutic intervention scoring system: the TISS-28 items–results from a multicenter study. Crit Care Med. 1996;24(1):64–73. 8565541 10.1097/00003246-199601000-00012

[20] Keene AR, Cullen DJ. Therapeutic intervention scoring system: update 1983. Crit Care Med. 1983;11(1):1–3. 6848305 10.1097/00003246-198301000-00001

[21] Reis Miranda D, Moreno R, Iapichino G. Nine equivalents of nursing manpower use score (NEMS). Intensive Care Med. 1997;23(7):760–5. 9290990 10.1007/s001340050406

[22] Miranda DR, Nap R, de Rijk A, Schaufeli W, Iapichino G, Group tmotTW. Nursing activities score. Crit Care Med. 2003;31(2):374–82. 12576939 10.1097/01.CCM.0000045567.78801.CC

[23] Hoogendoorn ME, Brinkman S, Spijkstra JJ, Bosman RJ, Margadant CC, Haringman J, et al. The objective nursing workload and perceived nursing workload in intensive care units: analysis of association. Int J Nurs Stud. 2021;114(7):103852. 33360666 10.1016/j.ijnurstu.2020.103852

[24] Ivziku D, Gualandi R, Ferramosca FMP, Lommi M, Diaz MYT, Raffaele B, et al. Decoding nursing job demands: a multicenter cross-sectional descriptive study assessing nursing workload in hospital medical-surgical wards. Sage Open Nurs. 2024;10:23779608241258564. 38836188 10.1177/23779608241258564 PMC11149452

[25] Buestan M, Perez C. Identification of predictive nursing workload factors: a six Sigma approach. Sustainability. 2022;14(20):13169.

[26] Jackson J, Anderson JE, Maben J. What is nursing work? A meta-narrative review and integrated framework. Int J Nurs Stud. 2021;122:103944. 34325358 10.1016/j.ijnurstu.2021.103944

[27] Polit DF, Beck CT. Nursing research: generating and assessing evidence for nursing practice. 10th ed. Philadelphia, PA: Wolters Kluwer; 2017.

[28] Saunders B, Sim J, Kingstone T, Baker S, Waterfield J, Bartlam B, et al. Saturation in qualitative research: exploring its conceptualization and operationalization. Qual Quant. 2018;52(4):1893–907. 29937585 10.1007/s11135-017-0574-8 PMC5993836

[29] Hsieh HF, Shannon SE. Three approaches to qualitative content analysis. Qual Health Res. 2005;15(9):1277–88. 16204405 10.1177/1049732305276687

[30] Fagerström L, Vainikainen P. Nurses’ experiences of nonpatient factors that affect nursing workload: a study of the PAONCIL instrument’s nonpatient factors. Nurs Res Pract. 2014;2014:167674. 25050179 10.1155/2014/167674 PMC4090478

[31] McDermott C. Reimagining the preceptor role. Nurs Admin Q. 2023;47(3):227–33. 10.1097/NAQ.0000000000000580 37261411

[32] Carayon P, Alvarado CJ. Workload and patient safety among critical care nurses. Crit Care Nurs Clin North Am. 2007;19(2):121–9. 17512468 10.1016/j.ccell.2007.02.001

[33] Liu Q, Lu Y, Zhai S, Dai C, Kan C, Chen C. Workflow interruptions, perceived workload and missed nursing: their impact on nurses’ health status—a structural equation model. J Adv Nurs. 2025;0:0–10. 10.1111/jan.16855 39991959

[34] Havaei F, MacPhee M, Ma A, Wong VW, Li C, Cheung I, et al. Implementation of the synergy tool: a potential intervention to relieve health care worker burnout. Int J Environ Res Public Health. 2023;20(1):489. 10.3390/ijerph20010489 PMC9819893 36612811

[35] Mandal L, Seethalakshmi A, Rajendrababu A. Rationing of nursing care, a deviation from holistic nursing: a systematic review. Nurs Phil. 2020;21(1):12257. 10.1111/nup.12257 31429179

