# Supplementary material for: “They don’t know what it’s really like:” qualitative insights into inpatient cardiac nurses’ perceived workload
Source: BMC Nurs. 2025 Aug 18;24:1083. doi: 10.1186/s12912-025-03723-4 (PMC12363037; doi:10.1186/s12912-025-03723-4)
Supplement: Supplementary file 1 — Supplementary Material 1 [file 12912_2025_3723_MOESM1_ESM.docx]

**Introduction**

Hello everyone. Thank you for being here today.

***Purpose***: The purpose of this research study is to better understand nursing workload. Our hope is to identify the factors that shape nurses’ workload so that we can ultimately measure it better.

1. First, can you please tell me about your role and level of experience?
2. We’re trying to understand what contributes to high nursing workloads. So, I’d like you first to take a moment and recall a recent day that you had that was particularly challenging. Or perhaps, think about a day that sticks out in your mind as one of our worst days at work. *(pause)* Can you tell me what made that day so difficult?
   1. What types of tasks did you have to complete that day?
   2. What were your patients like that day?
   3. Tell me about your interactions with other people on the unit that day.
3. Now, please recall a recent day at work that felt easy. Can you tell me about that day?
   1. What contributed to that positive experience?
4. Now let’s think about the specific tasks that you perform during a typical day. What tasks do you dread doing the most?
   1. Why do you think those tasks feel so challenging or undesirable?
   2. Alternatively, what tasks feel the easiest?
5. We’re also trying to learn more about how nurses make nurse-patient assignments. If you are involved in making nurse-patient assignments, can you describe that process and the extent to which you consider your nurses’ workloads?
   1. When you’re trying to consider nurses’ workloads, how are you measuring that? What are you thinking about?
   2. Do you use an acuity tool on your unit? In what ways do you find this tool helpful or unhelpful?
6. We’ve talked about a lot today. Is there anything else that you think is important to add to help us better understand nursing workload?
